# Supplementary material for: Anapole nanolasers for mode-locking and ultrafast pulse generation
Source: Nat Commun. 2017 May 31;8:15535. doi: 10.1038/ncomms15535 (PMC5460025; doi:10.1038/ncomms15535)
Supplement: Supplementary Information — Supplementary Figures, Supplementary Notes and Supplementary References [file ncomms15535-s1.pdf]

Supplementary Note 1: Effect of losses in the Anapole laser

The role of losses and dissipation in the formation of Anapole states is a subject of research interest, as recently discussed in [1]. Supplementary Fig. 2 shows the multipole decomposition of the Anapole in an ideal (i.e., lossless) nanodisk of InGaAs, while Fig. 2b shows the corresponding scattering cross section (blue solid line). The scattering cross section in the presence of losses (imaginary part of the refractive index k=0.15) is presented in Fig. 2b, orange solid line. As seen from Fig. 2b, the effect of losses is to slightly reduce the overall scattering, which maintains values very close to experimental results in Si and Ge semiconductors [2,3]. To provide a deeper analysis on the effect of losses on this structure, we calculated in Supplementary Fig. 3a-e the multipolar decomposition of the electromagnetic field at the Anapole frequency in the case of losses, with Fig. 3f showing the corresponding absorption cross section of the system. In the InGaAs nanodisk , as we see from Fig. 3, the net effect of losses in linear regime is to slightly reduce (of almost the same factor) the energy coupled into all multipolar components of the electromagnetic field. As shown in Fig. 3f, the energy difference that is not coupled to electromagnetic components contributes to increase the absorption scattering cross section of the nanodisk. In our particular case, this variation is little as the losses of the system are small. Supplementary Figure 4 shows FDTD simulations of the system linear response when a plane wave impinges on the nanodisk supporting an Anapole state in the (a) lossless and (b) real InGaAs medium with k=0.15. As we see from FDTD simulations, the linear response of the system is almost the same in both cases.
Quite interesting is the effect of losses in the Anapole laser, where the Anapole state is nonlinearly amplified via stimulated emission of radiation. We evaluate this effect by first calculating the energy distribution of an ideal anapole, presented in Supplementary Figure 5. This calculation is performed by extracting the anapole near field energy distribution from the scattered field from an ideal lossless InGaAs nanodisk with k=0. We then computed the cross correlation between the ideal Anapole of Supplementary Fig. 5 and the nonlinearly amplified Anapole shown in Fig. 3 of the main text and calculated for a realistic, lossy InGaAs structure with k=0.15. As observed in the main text, the cross correlation coefficient is extremely high and equal to 96.1%. This results from the fact that the electromagnetic losses, as shown in Fig. 3f, increase the amount of energy trapped inside the nanodisk. This mechanism does not alter the nature of the anapole state being amplified, as the latter is a radiantionless state whose energy exists mainly within the nanodisk. This result suggests also that nonlinear amplification of anapoles via stimulated emission is a viable solution to excite almost ideal anapole states in realistic lossy structures.

Supplementary Note 2: Nonlinear dynamics of the Anapole chain

In this section we derive a simplified model of light matter interaction to describe the main feature of the dynamics of the nonlinear Anapole chain and help interpreting the results arising from first-principle simulations. By generalizing the theory of light-matter interactions in coupled laser systems (see section II of [4]) to the case of multi-frequency lasing structures, we obtained the

following equations that describe the dynamics of the anapole chain:

$$\begin{cases} \dfrac{dE_n}{dt} = (1+i\alpha)N_n E_n + i\omega_n + \displaystyle\sum_m C_{nm} E_m, \\ \dfrac{dN_n}{dt} = \mu[P - N_n - (1+2N_n)|E_n|^2], \end{cases} \tag{1}$$

being E_n is the electric field amplitude of the the $n-$th Anapole in the chain, $t = t/\tau_{\mathrm{p}}$ a dimensionless time measured in units of the photon carrier lifetime τ_{p}, N_n the population inversion, α the linewidth enhancement factor, $\mu = \tau_{\mathrm{p}}/\tau_{\mathrm{s}}$ the ratio of photons τ_{p} to carrier τ_{s} lifetime, P the pumping rate and $C_{nm} = c_{mn}e^{i\psi_{nm}}$ coupling coefficients, arising from spatial overlaps between interacting Anapoles. The term $i\omega_n$ can be factored out by rescaling the electric fields $E_n \to E_n e^{i\omega_n t}$:

$$\begin{cases} \dfrac{dE_n}{dt} = (1+i\alpha)N_n E_n + \displaystyle\sum_m C_{nm} e^{i\Delta\omega_{mn} t} E_m, \\ \dfrac{dN_n}{dt} = \mu[P - N_n - (1+2N_n)|E_n|^2], \end{cases} \tag{2}$$

with $\Delta\omega_{mn} = \omega_m - \omega_n$ mismatched contributions. At leading order in the dynamical equations (2), only phase matched terms $\Delta\omega \sim 0$ contribute to the evolution of the system, while mismatched terms generate oscillating terms that are integrated out in time. In our Anapole chain, this condition applies only to nearest-neighbour Anapoles, which have weakly overlapping energy distribution via exponential tails and frequencies very close in the spectrum (spacing of only few nm). This condition allows to simplify the dynamics as follows:

$$\begin{cases} \dfrac{dE_n}{dt} = (1+i\alpha)N_n E_n + \displaystyle\sum_m C_{nm} E_m, \\ \dfrac{dN_n}{dt} = \mu[P - N_n - (1+2N_n)|E_n|^2], \end{cases} \tag{3}$$

With nearest neighbor nonzero coupling elements C_{nm}.

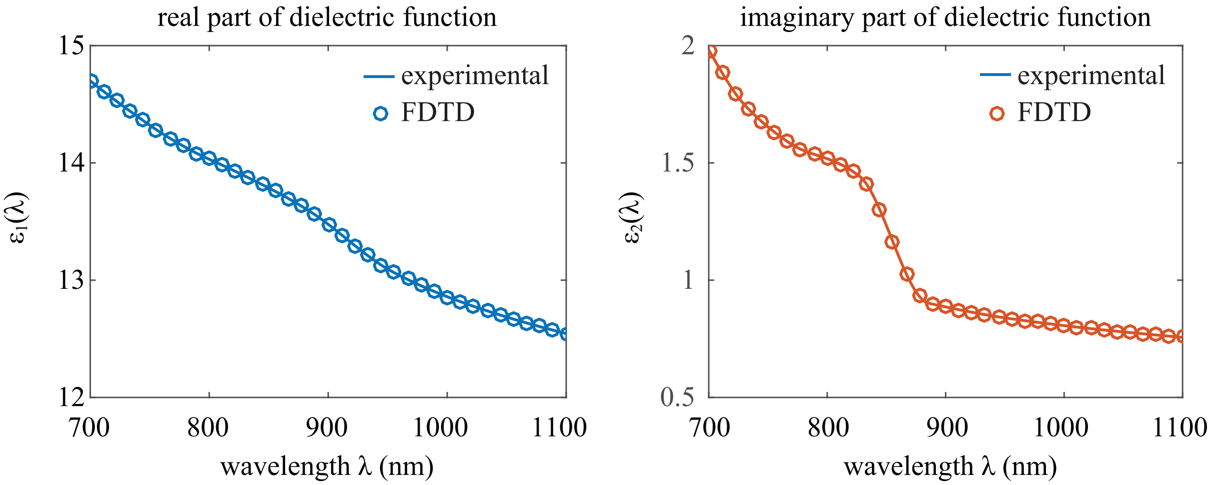

Supplementary Figure 1: Dispersive FDTD modeling of $In_{0.15}Ga_{0.85}As$ Real (left) and imaginary (right) parts of the dielectric function $\epsilon(\omega) = \epsilon_1(\omega) + i\epsilon_2(\omega)$ of $In_{0.15}Ga_{0.85}As$ as a function of the wavelength λ. The dispersion model employed in our simulations (circles markers) ensures one-to-one mapping with experimental measurements (solid lines).

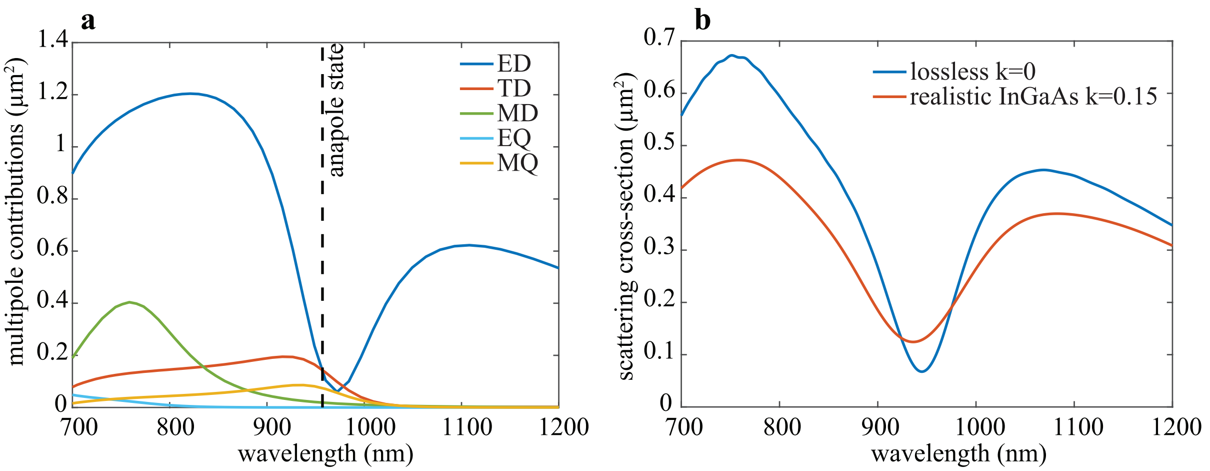

Supplementary Figure 2: a Multipole analysis of an ideal anapole state. The multipole components are computed in Cartesian coordinates and they correspond to: Electric Dipole (blue line), Toroidal Dipole (orange line), Magnetic Dipole (green line), Electric Quadrupole (cyan line), Magnetic Quadrupole (violet line). At the anapole frequency (dashed vertical line), toroidal and electric dipole cancel each other producing the radiation-less state. **b** Scattering cross-section for an $In_{0.15}Ga_{0.85}As$ nanodisk of diameter $d = 440$ nm and height $h = 100$ nm in an ideal lossless configuration (k=0, blue line) and in the realistic lossy configuration (k=0.15, orange line) .

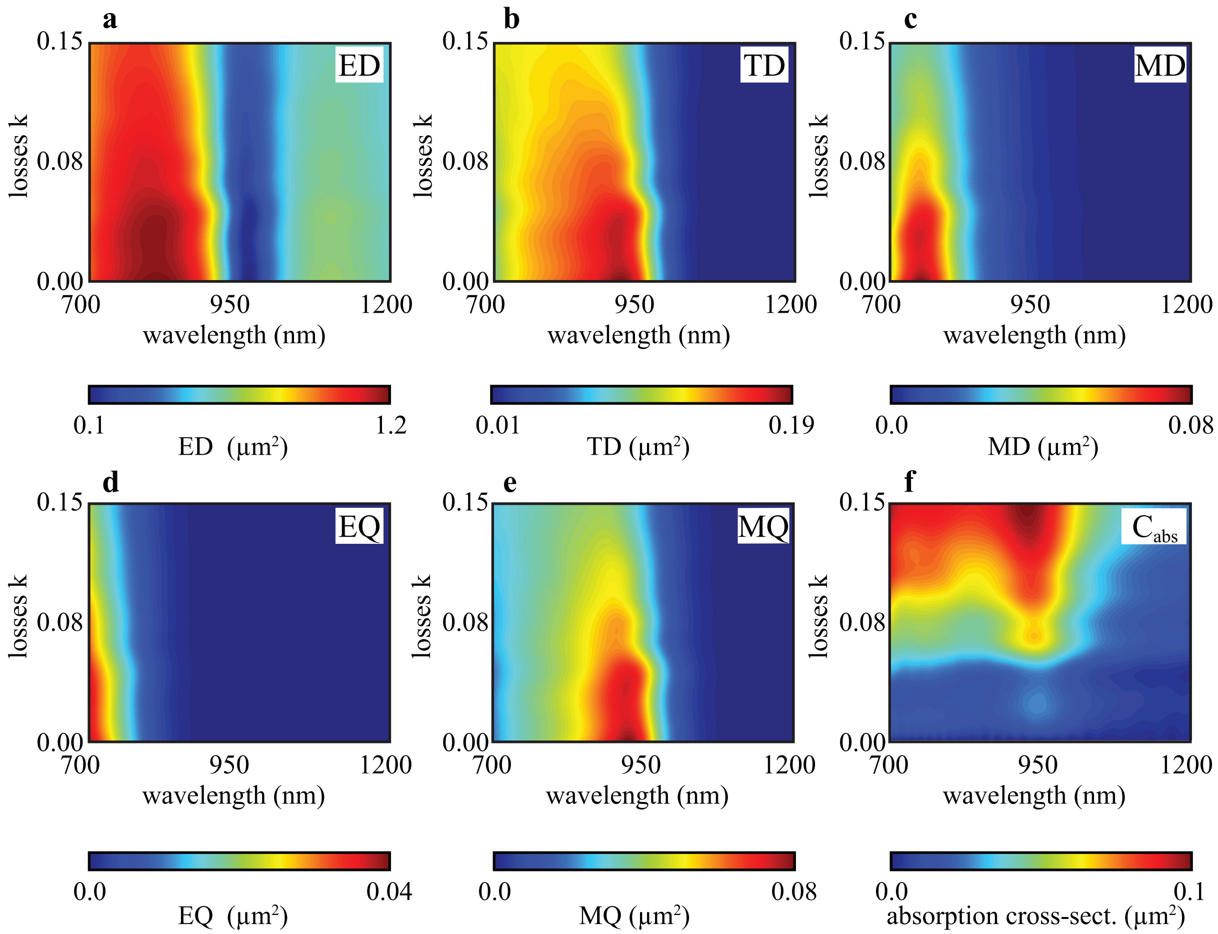

Supplementary Figure 3: Effect of losses in the anapole laser. a-e Multipole decomposition for an InGaAs cylinder of height h=100 nm and radius 220 nm as a function of the incident wavelength and the imaginary part of the refractive index k. The multipole components are: (a) Electric Dipole, (b) Toroidal Dipole, (c) Magnetic Dipole, (d) Electric Quadrupole and (e) Magnetic Quadrupole. **f** Absorption cross-section.

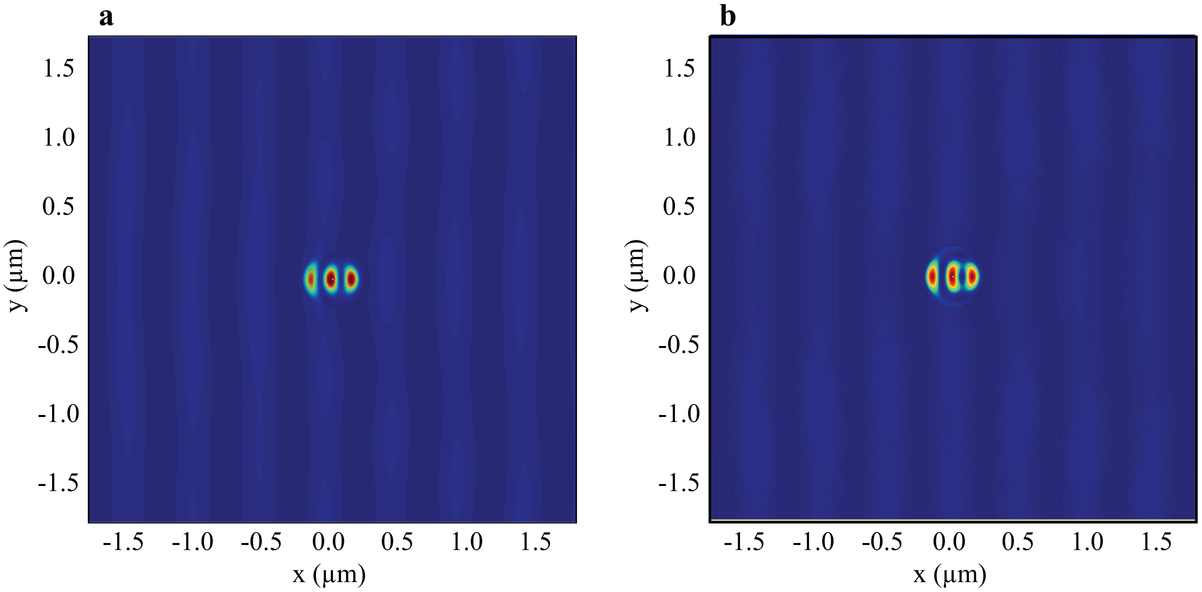

Supplementary Figure 4: Effect of losses and scattering suppression of an anapole state. **a,b** Electromagnetic energy distribution for an $\mathrm{In}_{0.15}\mathrm{Ga}_{0.85}\mathrm{As}$ disc of diameter $d = 440$ nm at the anapole wavelength $\lambda = 498nm$. The difference between the (a) lossless and (b) realistic lossy configuration is minimal. The imaginary refractive index is (a) $k = 0$ and (b) $k = 0.15$.

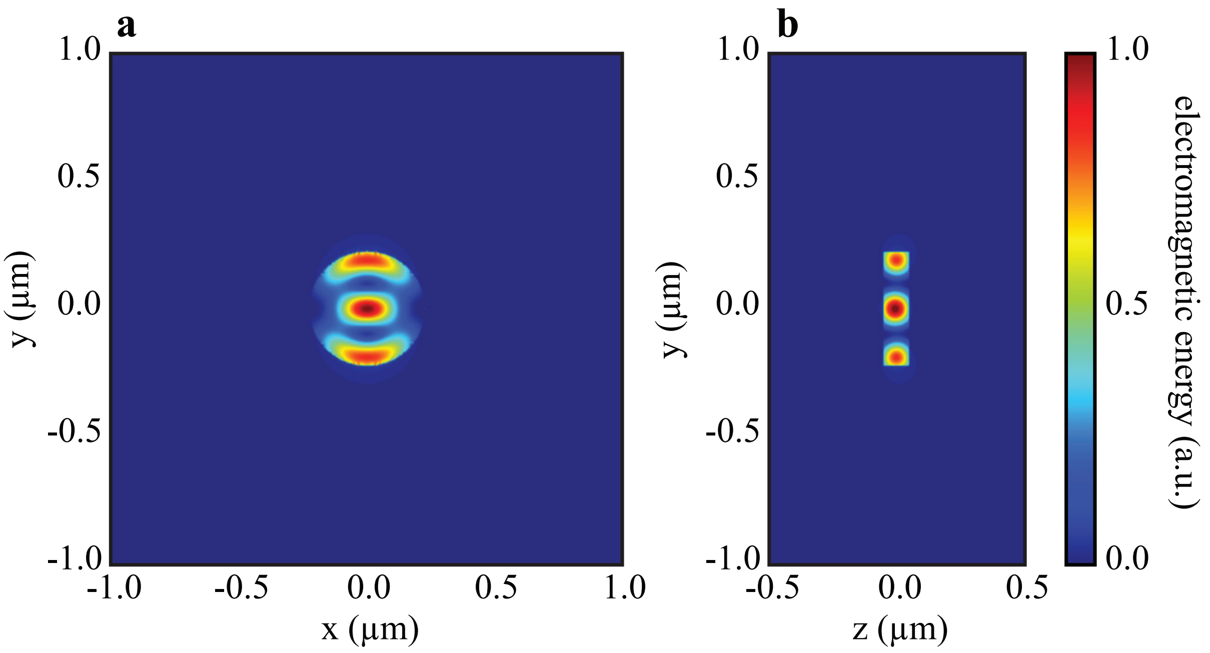

Supplementary Figure 5: Comparison between the anapole laser emission and an ideal anapole state. **a,b** Scattered Electromagnetic energy from an Ideal anapole ($k = 0$) for an $In_{0.15}Ga_{0.85}As$ nanodisc of diameter $d = 440$ nm and height $h = 100$ nm. The sections correspond to the (a) $z = 0$ and (b) $x = 0$ planes, respectively. The three-dimensional spatial cross-correlation X_c with the Anapole laser (cfr. Fig. 3 of the main text) is $X_c = 0.961$.

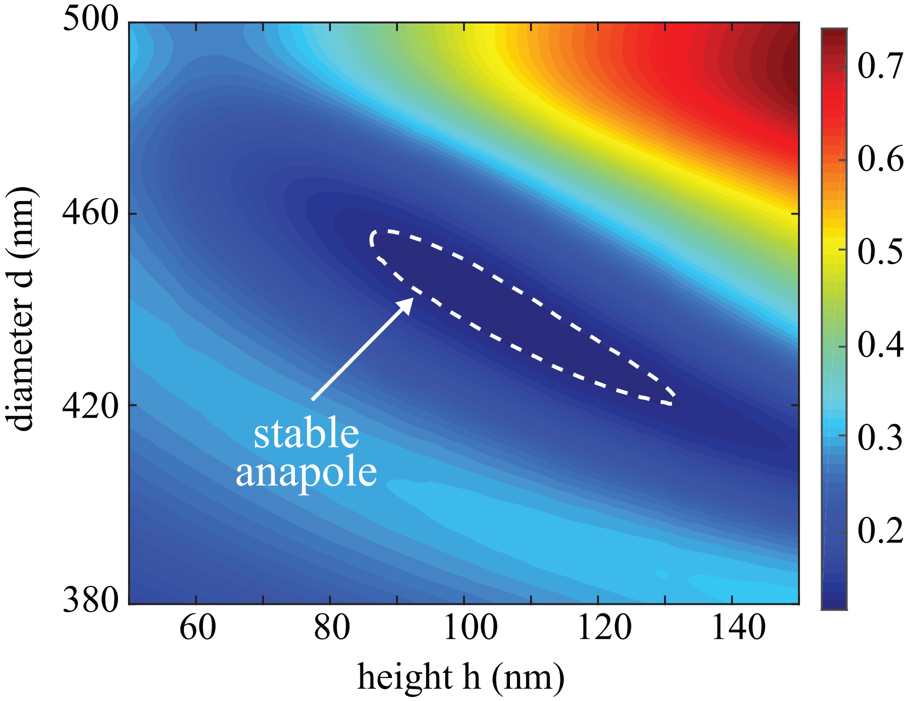

Supplementary Figure 6: Optimization and robusteness of the anapole state at $\lambda = 950$nm. Scattering cross section for an $In_{0.15}Ga_{0.85}As$ cylinder for varying diameter and height near the anapole state formed at $d = 440$ nm and $h = 100$ nm. The dotted area shows the region of strong scattering suppression, where the anapole state is stable.

a

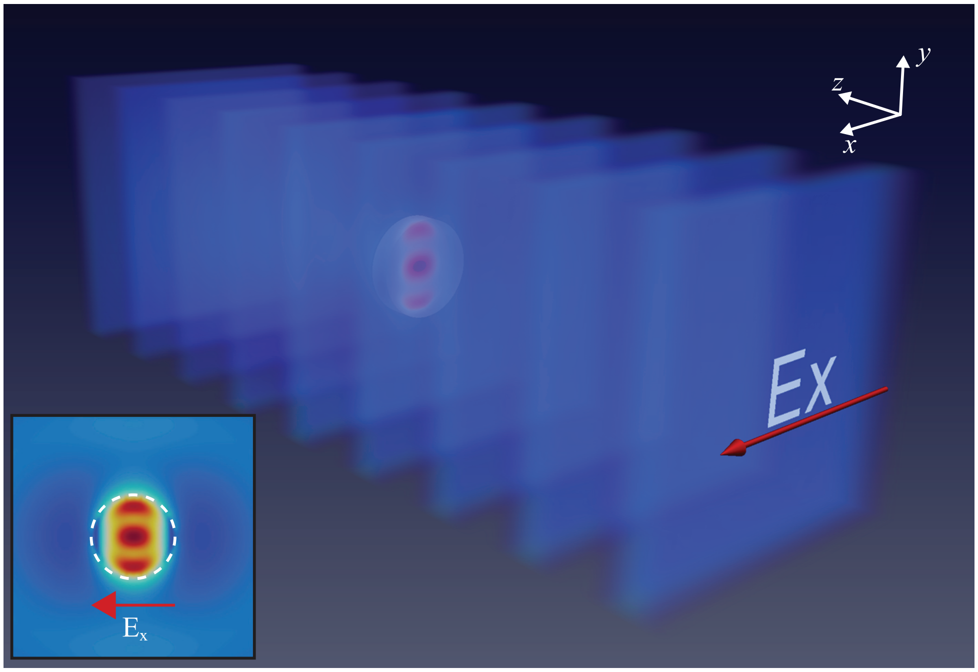

b

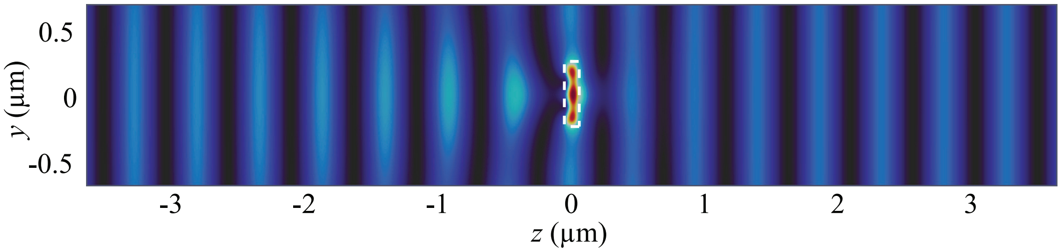

Supplementary Figure 7: Scattering suppression and invisibility of a linear anapole state. a Volume mapping of the electromagnetic energy distribution of an incident plane-wave impinging on an anapole nanoparticle at $\lambda = 948$nm. The plane wave propagates along z, and the incident polarization is E_x (red arrow). (inset) Section of the electromagnetic energy in the center of the cylinder (dashed line), showing the characteristic three-lobes profile of the anapole mode. **b** $x - z$ section of the electromagnetic energy distribution. As can be seen from the figure, the anapole scattering field is restricted to the near field, and the plane wave profile is completely restored after a few wavelengths.

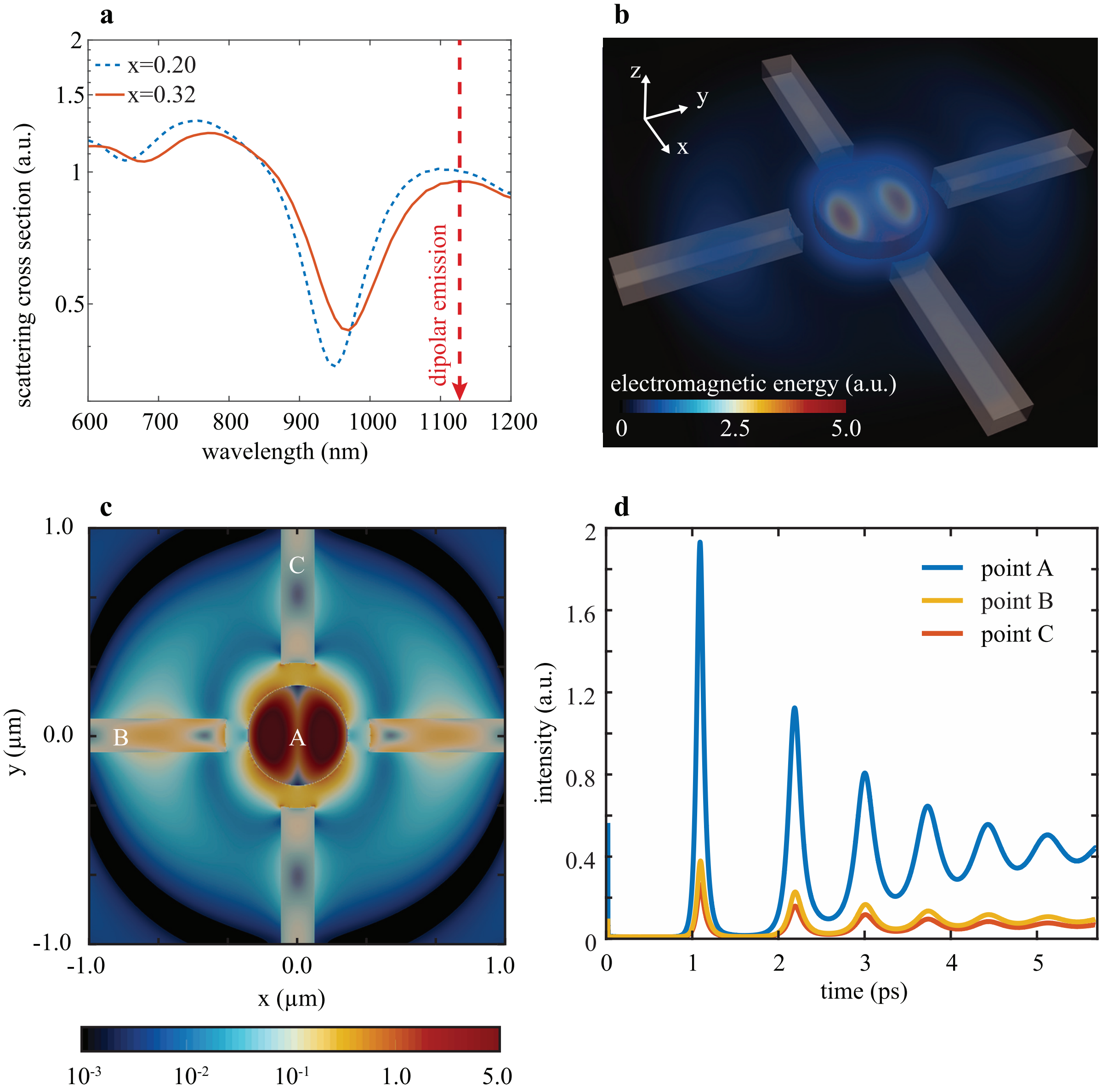

Supplementary Figure 8: Dipolar emission from $In_{0.32}Ga_{0.79}As$ nanodisk. **a** Scattering cross section from a cylinder with height $h = 100$ nm and $d = 440$ nm composed of $In_{0.15}Ga_{0.85}As$ (dotted blue line) and $In_{0.32}Ga_{0.79}As$ (solid orange line). The emission of $In_{0.32}Ga_{0.79}As$ occurrs at 1125nm (red arrow), corresponding to a scattering peak of the structure. **b** Volumetric plot of the electromagnetic energy corresponding to the dipolar emission at $\lambda = 1195$nm. **c** (x, y) section of the electromagnetic energy. The pseudo-color plot is based on a logarithmic color scale. **d** Electric field intensity in the resonator (blue line, point A of panel c), in the x-parallel waveguide (yellow line, point B of panel c) and in the y-parallel waveguide (orange line, point C of panel c).

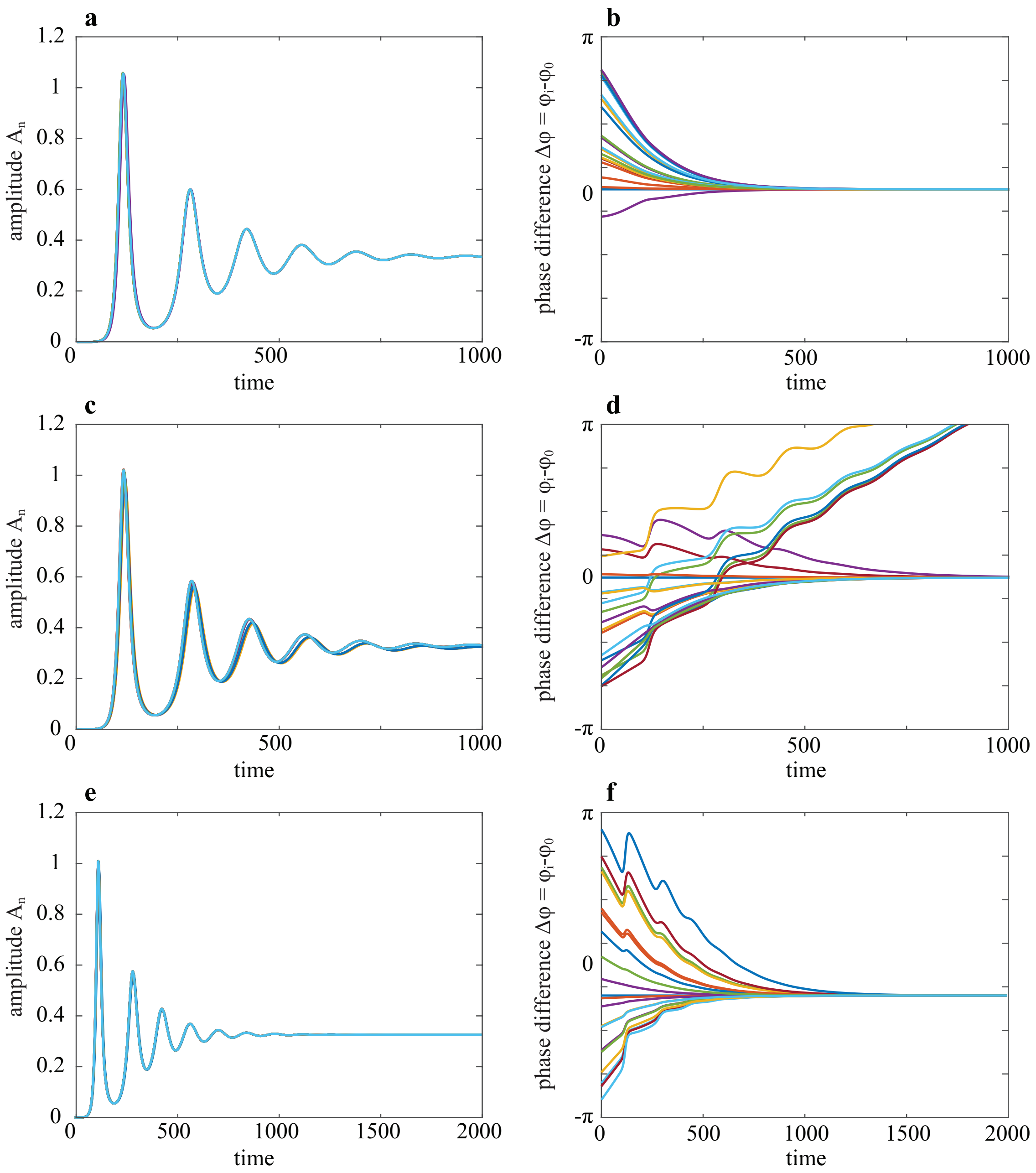

Supplementary Figure 9: Nonlinear dynamics in a chain of anapole lasers Modal amplitudes $A_n(t)$ (left panels) and phase differences $\Delta\varphi_n(t) = \varphi_n(t) - \varphi_0(t)$ (right panels) as obtained by numerical integration of Eqs. (3). In the case $\alpha = 0$, the system is a Kuramoto model, where (a) amplitudes and (b) phases lock in time. When $\alpha = 1$ only a few modes interact and synchronize (panels c and d). The overall locking can be restored by acting on the coupling matrix C_{mn}: in the case of an self-adjoint coupling matrix, in fact, the phases synchronize on a longer time scale. Simulation parameters: $\mu = 0.01$ and $P = 0.1$. In panels a-d, the coupling matrix C_{mn} is a random tridiagonal matrix, while panels e and f the random coupling matrix $\mathbf{C}$ is imposed to be self adjoint (i.e., by imposing $\mathbf{C} = \frac{1}{2}(\mathbf{A} + \mathbf{A}^{\dagger})$, being $\mathbf{A}$ a random tridiagonal matrix). The initial phases of the system are random values in $[0, 2\pi]$.

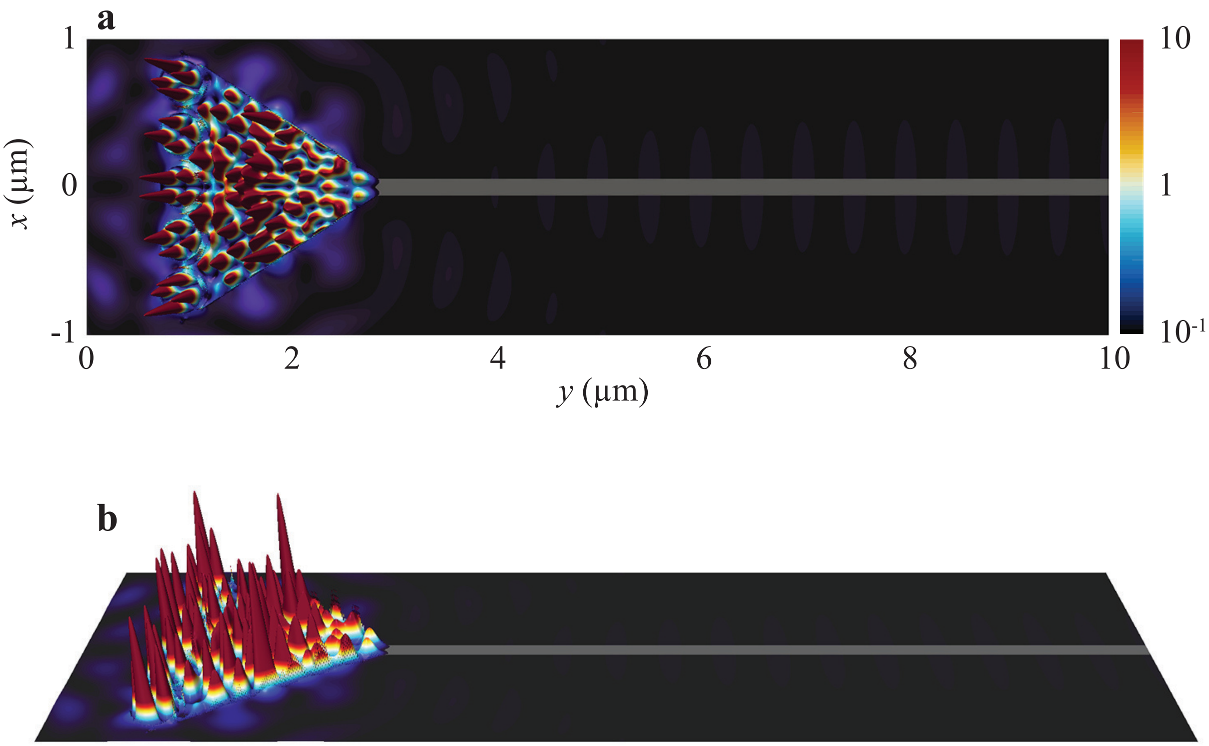

Supplementary Figure 10: Emission from an array of highly-scattering modes. a Surface plot of the (x-z) section of the electromagnetic energy of the same structure as in Fig. 5 of the main text but in the presence of highly-scattering nanoparticles. The nanoparticle radius is $r =$ 203nm, which corresponds to a scattering peak at the emission wavelength λ_0. The emission of the nanoparticles is coupled into the tapered structure but fails to produce a propagating mode in the waveguide (solid gray). **b** Tilted view of (a).

Supplementary References

1. Tribelsky, M. I. & Miroshnichenko, A. E. Giant in-particle field concentration and Fano resonances at light scattering by high-refractive-index particles. *Phys. Rev. A* **93**, 053837 (2016).

2. Miroshnichenko, A. E. *et al.* Nonradiating anapole modes in dielectric nanoparticles. *Nat. Commun.* **6**, 8069 (2015).

3. Grinblat, G., Li, Y., Nielsen, M. P., Oulton, R. F. & Maier, S. A. Enhanced Third Harmonic Generation in Single Germanium Nanodisks Excited at the Anapole Mode. *Nano Lett.* **16**, 4635–4640 (2016).

4. Javaloyes, J., Mandel, P. & Pieroux, D. Dynamical properties of lasers coupled face to face. *Phys. Rev. E* **67**, 036201 (2003).
